# A Treatise on Those Diseases of the Brain and Nervous System Which Are Usually Considered and Called Mental

**Published:** 1833-10

**Authors:** 


*A Treatise on those Disorders of the Brain and Nervous System which are usually considered and called Mental.* By David Uwins, M.D. London, 1833. 8vo. pp. 235.

Dr. Uwins has here presented us with a readable, but by no means profound, treatise on the diagnosis and treatment of Insanity. In the first chapter, which is headed "Preliminary,—Nosology, Phrenology, and Nomenclature," our author is exceedingly vague and unsatisfactory, and rambles on for ever in the following strain:

"As on all hands, and by all theorists, it is admitted that the brain and nerves are in some mode or other the media of perception and consciousness, writers on madness have evinced an anxiety to acquire a knowledge of the laws by which these organs and their functions are regulated, on this ground,—that, could we ascertain the precise circumstances of mental health, we should be better qualified to mark out and map those manifold deviations from it to which all are obnoxious. But philosophers and physiologists, in their researches on this head, have all egregiously failed. The ποῦ στω has ever eluded the research of the metaphysician, so much so, that the reader of these pages would be but ill repaid for his trouble, were I to attempt any thing like a detail of successive doctrines: and, if metaphysicians have been bad, medico-metaphysicians have been still worse. '*All* medical doctrines,' says Dr. James Gregory, in his blunt and forcible manner, 'are stark staring nonsense;" and it cannot be denied, that the disputations of the schools afford considerable justification to this sweeping condemnation of pathological theory. Thought, says one writer, is a secretion from the brain, as bile is from the liver. Ideas, says another, are vibrations and vibratiuncles. A third calls them configurations of the organs of sense. These are all modern, very modern conceits, and the individuals who have broached them are men of high intellect, and of moral worth. In what, however, do the conceits themselves differ from, but in their being more vague and unphilosophical than the phantasms of Plato, the Pineal gland of Descartes, and the Archæus of Helmont?

"Whence all this nothingness of abstract speculation? Why it is because the inquiries have been abstract, that they have so lamentably failed. The essential distinction has been lost sight of between final and efficient cause. A point has been assumed, where matter ends, and mind begins; analogies have been eagerly sought for, while it has not been duly considered that the very terms mind and matter are gratuitous, and in nowise expressive of essence. Thus philosophy has been made a species of poetry, to 'airy nothings' have been given 'habitations and names,' and the whole fabric of medico-metaphysical speculation comes out to be merely formed of 'the stuff which dreams are made of.'

"Has the new organology at all assisted our extrication from these

'muddy depths of everlasting nonsense?' Have they sent physiological and pathological researches into the right path of pursuit? Have Gall or Spurzheim thrown out any intimations respecting mind, or rather its media, which may prove at last *ad rem?* or do these psycologists still bind us in those delusive tracks, which lead to that delightfully dread abode, the Castle of Doubt, 'delightfully,' O, yes! I wish, when thinking of it, to be young again, in order to enjoy its picturesque beauty, as magnificently pourtrayed by its great delineator, with the appendages of its grim ruler, Giant Despair, and the restless, uxorious upbraiding of his anti-christian and anti-hopeful wife; but, '*tempora mutantur*, and, alas! *nos mutamur*.'

"In attempting a reply to the above questions, I expect it will be my fate not to satisfy either party. There is still, to my seeming, a great deal of mental circumstance quite inexplicable upon, if not in opposition to, the tenets of the phrenologists; but at the same time I am of opinion, that to the ingenious theorists just named, physiology is much indebted." (P. 13.)

In the second chapter, entitled "Definition, Essentials," Dr. Uwins is still harping on the utter impossibility of defining insanity, and says,

"In respect to the kinds and shapes of it as marked out by authors, the reader will already have perceived that I do not attach much value to them. *Melancholia*, why we are all melancholic at times from misconception. *Mania*, who is there that has not been at one period or another irritated beyond measure by misapprehension? *Monomania*, or misconception on one point. What light do we throw upon this feeling by giving it a hard name! Of *Hypochondriasis* we have plenty of instances in the present day, much short of mad-house dreamers, since the stomach has been considered every thing, and every thing the stomach. Even *Idiocy* may be monoidiotism, as seems to have been the case in the Baxter instance; and we are all more or less idiotic, if the term implies congenital want of power. An Oxford professor, and an erudite man too, attempted again and again, but without success, to learn the art and mystery of whist-playing; it was no want of disposition that occasioned the want of capacity, because his earnest desire was to acquire a knowledge and facility in the game. An angel from heaven, says Fuseli, could never give me the comprehension of mathematics; and every one has heard of the philosopher's inquiry, after having read Milton's epic from beginning to end, '*Cui bono*? What does it all mean? What does it *prove*?'

"*Dementia* is the idiocy of old age or of accident, or of overworking, or of letting lie altogether fallow the mental powers, and the same objections may be taken, as in the other cases, to the specification of it as an essence; for I still repeat my conviction, that there is much more propriety in saying a man is so idiotic, or so stupid, or so mindless, or so melancholic, or so mad, as to render it necessary that he should be deprived of the right of a rational creature; than imputing any specific or essential disorder, as a something coming upon an individual in a wholesale or abstract way:

> ——*ου γὰρ ἀύτος πάντ᾽ ἐπίστασθαι Βροτῶν*
> *Πέφυκεν· ἄλλῳ δ᾽ ἄλλο πρόσκειται γέρας.*"—

(P. 32.)

The answer to all this is so very obvious, that we are almost ashamed of making it, especially as Dr. Uwins has himself nearly hit upon it in the latter part of the above quotation. Although, in philosophy, *majus et minus non variant speciem*, yet in nosology, as in common life, we are obliged to admit the unphilosophical distinction; and a high fever is as different a thing from a slight one as a tall man is from a short one, though it is allowed on all hands that it is impossible to define how many feet and inches make up a giant, or what intensity of symptoms will just constitute a hopeless case of typhus. Instead therefore of saying, with our author, that a man is so mad, or so stupid, &c. that he ought to be shut up, we prefer saying, with the rest of the world, that he is mad; and content ourselves with applying the terms eccentricity, melancholy, &c. to slighter deviations from ordinary behaviour, or ordinary ways of thinking. We should define madness to be *such a deviation from the usual theories or practice of mankind as does not carry with it the sympathy of any class of human beings.* The most obvious cases of all are those in which the patient has either lost the consciousness of his own personal identity, or of the nature of the common objects by which he is surrounded, like the man mentioned by our author who took a piece of string to be a letter from his wife; and we might proceed gradually through less and less obvious instances, till we came to persons who are adjudged to be insane by those in a different rank of life, or of a different frame of mind, though their habits are perfectly natural in their own sphere. Thus we recollect that Davies, the tea-dealer, was pronounced, by a grave and learned doctor of physic, to have exhibited a certain proof of insanity in withdrawing a few thousand pounds from his trade, in order to buy a small estate in the country.

Dr. Uwins having got over this difficult definition as well as he can, proceeds in the next chapter to give what he calls "Progress of Symptoms; General Characteristics." On the progress of the symptoms he has nothing at all, except a bit quoted from Haslam; but on the other branch of the chapter he is more diffuse, and has some pertinent remarks; as, for example, the following:

"One prominent characteristic of derangement is an offensive breath, by which, it has been remarked, you may distinguish between real and feigned disorder. This symptom, it may be, in the general way, is too loosely taken as a proof of stomach affection, for the confirmed lunatic will eat, and drink, and digest with facility, at the time that the above accompaniment to deranged consciousness is most conspicuous. The passions of the mind will immediately produce these changes in pulmonary exhalations, without disorder going through the course of the first passages.

"The talkative lunatic is remarkable for the repetition of a word that may have been accidentally used by himself, or expressed by another; and such repetition is, for the most part, still more persisted in, despite of endeavours of bystanders to supersede it, should it fall into a sort of rhythm with other parts of a sentence. On the other hand, what has been called by Shakspeare re-wording can seldom be accomplished by an individual whose ideas are jumbled together insanely. This difficulty in distinctly repeating a proposition ought to be recollected by all who are professionally called on to decide mental conditions, as it is extremely significant of derangement. It was alluded to particularly in a paper read before the College of Physicians, some time since, by the president.

"Madmen are usually considered to be insensible to the lapse of time, past and to come being all resolved into present perception; and there is some correctness in the notion. What an affecting illustration of this *eternal now* of the maniac, is that which Mr. Hill presents us with, in his work on Insanity.

"'A gentleman, on the point of marriage, left his intended bride for a short time; he usually travelled in the stage-coach to the place of her abode; the last journey he took from her was the last of his life. Anxiously expecting his return, she went to meet the vehicle. An old friend announced to her the death of her lover. She uttered an involuntary scream, and piteous exclamation "he is dead!" From this fatal moment, *for fifty years*, has this unfortunate female daily, in all seasons, traversed the distance of a few miles, to the spot where she expected her future husband to alight from the coach, uttering in a plaintive tone, "He is not come yet; I will return to-morrow."'

"In this particular, however, as in almost all others, there is much of irregularity and inconsistency. I shall most probably on this day be asked, by many of my Peckham patients, "Pray, sir, when do you intend to let me out of this place? Here I have been confined for—' so many years, and weeks, and days; and, in comparing notes, I find their accounts correct. But some of these very patients, in other things, will show a want of proper perception of the lapse of time; manifesting the same inconsistency with a consumptive girl, who shall at one hour talk of shortly dying, and in the next will be ordering changes of dresses, to meet the changes of fashion that have taken place during her protracted sickness.

"An insensibility to cold, and heat, and disease, in their extremes, has been pronounced peculiar to insanity; but, although in some cases the intensity of the predominating feeling makes the madman either careless of or insensible to other impressions, you will, for the most part, see lunatics crowd round the fire on winter days, exactly as would the inmates of other establishments. Cholera, too, swept several of our worst cases out of the world; and many were affected with influenza.

"Again, some maniacs will refuse sustenance with obstinacy, while others will take it with avidity. Dr. Reid remarks, that 'hypochondriasis has often to thank calamity for its cure.' Certain it is, that dyspepsia in many cases flies away from mania. I remember the case of an intimate friend and interesting patient, who, for years previous to that extent of disorder which called for interference, was scarcely able to eat or drink anything with impunity, afterwards took his meals, of whatever they might consist, without the smallest hesitation, and without the slightest stomach disturbance. It is probable, I think, in this instance, that, had not madness succeeded dyspepsia, some organic derangement of the digestive organs might have done so instead; and I have often thought that, had Napoleon been suffered to continue his career of ambition, madness might have visited him, rather than cancer of the pylorus.' (P. 42.)

In the fourth chapter, on the "Sources of Insanity," there is little to detain us. Love, methodism, spirit-drinking, opium-eating, and intense study, are all very naturally mentioned as causes of insanity; and so is even tea-drinking; not so much, however, as Dr. Willis thought, physically, but rather morally.

"It is not the mere abstract poison of tea which deteriorates the nervous system, (though there is something even in this;) but it is the accompaniments which tea brings with it that do the greatest part of the mischief. Pianos, parasols, Edinburgh Reviews, and Paris-going desires, are now found among a class of persons who formerly thought these things belonged to a different race: these are the true sources of nervousness and mental ailments, and not merely this or that specific article of food or drink." (P. 51.)

In fact, the general diffusion of tea is the index of the diffusion of refinement, and perhaps it may be true that, when great refinement and small means are found in company, the deepest melancholy, or madness itself, will often be the result. There is another, and a more cheerful side, however, to this picture. The growing taste for tea, as Mr. Rush observes, in his late work on England, must tend to check the passion for spirits, and thus cut off one of the most fertile sources of mental and bodily disease. A truly philanthropic chancellor of the exchequer would make souchong cheap, and gin dear.

Dr. Uwins, who appears, by-the-bye, to have a slight

*penchant* for paradoxes, proceeds to declare, in the following passage, that the books on disorders have multiplied the ailments which they profess to cure, so that young ladies fall into hysterics, and old ones set on foot a carcinoma uteri, because every street has its ladies' doctor ready to relieve them.

"It is a curious fact, and it being mentioned here may serve to strengthen my assumption, that the multiplication of rules about diet and regimen brings with it an increase of the very evil that is so anxiously sought to be avoided. When did dyspepsia prevail so hugely as it does at this moment, when we have treatise upon treatise, and precept upon precept, on stomach complaints. Who ever heard of such a numerous host of heart affections as now exist, now that the very vulgar talk largely and learnedly about valves and ventricles, and functions and organs? Do not teeth disorders increase in number with the multiplication of dentists, even of science and principle? and are not female complaints manifestly more numerous and complicated, now that there is an obstetrician in every street?" (P. 51.)

In the next chapter, which treats of the "Pathological Conditions, or Proximate Causes," Dr. Uwins very justly observes, that it is by no means certain that inflammation is the essence of mania; and he concurs with Willis, he says, in endeavouring to check that *mania* for lowering which many of our pathologists are addicted to. In this, as well as in other divisions of our author's work, we recognize the presence of misplaced passages, giving the book rather the appearance of notes for a treatise, than of an actual and digested treatise. The following fragment, on the return of memory, though in itself well put together, seems to us to have no connexion whatever with the proximate causes of insanity.

"Who shall explain, upon principles at all recognizable as legitimate, the circumstance of latent and unconscious knowledge lying in the brain, as it were, and only called out by accidental or adventitious circumstance? I remember, when a student at St. Thomas's Hospital, the case of a man who came from Gibraltar, supposed to be deaf, either from inattention or ignorance on the part of the surgeons who examined him. This man, it was ascertained, was labouring under compressed brain, in consequence, it is supposed, of a fall. Mr. Cline performed the operation of lifting up the part of bone which was thus by its pressure interfering with brain function, or with its manifestation rather, and the moment the pressure was removed, the poor man began talking Welsh, a language which he knew when a boy, but which he had forgotten in after-years; and we occasionally hear of cases very similar. The much talked of phenomenon of 'light before death' is derided by some, but the derision is misplaced; and when, I have elsewhere said, it is exultingly asked by the sceptic respecting a paralysed individual, where is the immortal mind which was wont to animate the features and guide the conduct of this man, now reduced to a state of mere animal vitality? we might appeal to instances, and they are not few, in which, just prior to the period of a total extinction of the living principle, the soul seems to come out from its hiding-place, and to cast a parting glance at the surrounding scene, as the sun often sinks bright and glorious below the horizon after having been the whole of the day obscured by clouds. Who has not dwelt with agonizing delight on the transcendently beautiful representation of Mrs. Opie, in her inimitable work entitled 'Father and Daughter,' where she makes the return of reason in the distracted parent the signal that all is about to close with him, so far as this world is concerned? and the delineation is no less true to nature than it is impressive and affecting. Something of the same kind happened in the case of my own father, who had been unconscious for years; and my respected friend and late colleague, Dr. Hancock, related once to me the account of a most respectable individual, belonging to the Society of Friends, who had been for a very long time deprived of his faculties by a stroke of palsy; nay, to use Dr. H.'s own words, who had been for this lengthened period in a state of drivelling idiocy, but who, for some time previous to his death, was restored to the full possession of his rational powers; he summoned his astonished family around him, delivered to each of them his parting advice and benediction, and then calmly resigned himself to a peaceful death!" (P. 78.)

The sixth chapter, on the "Prospects of Recovery, or Threats of Permanence," begins with the following curious defence of its own title:

"Medical terms are for the most part barbarous; and, so far as it is at all consistent with the nature and design of the present traetise, I am disposed to avoid them. It is, however, much easier to find fault than to find remedies; and it is of course requisite to devise some leading words, in order to embody, as much as may be, a general principle, or a series of circumstances. I could not, however, persuade myself to write prognosis at the top of the present page; and I take occasion here to intimate, that, were medical authors to supersede, as much as possible, a harsh terminology, and 'exhibit medicine in the simplicity of nature, and the nakedness of truth,' they would be likely to command more unreserved respect, and be listened to with greater attention." (P. 88.)

Now, if some kind friend had suggested that "a *sine quâ non* medicinal" (p. 223,) was a barbarism for "an indispensable medicine," or if some critic, less lenient than ourselves, had insinuated that "the matter of an abscess" (p. 86, *note*,)

was an awkward periphrasis for *pus,* we should not have been surprised; but we confess we are astonished at finding *prognosis* called barbarous, though it has been in constant use from the days of the Father of Physic to the publication of this book, a period of two thousand and some odd hundred years. We would bet a Princeps Hippocrates against a copy of Dr. Uwins's Treatise, that the synonyme devised by him, namely, *prospects of recovery, or threats of permanence,* will not supplant PROGNOSIS.

But to return from words to things. The prognosis is unfavourable when the disease is hereditary, when the unhappy patient obstinately clings to some one false notion, or when he is epileptic. Indeed, the last fact is so well established, "that our two great institutions, Bethlem and St. Luke's, in a spirit of cruel policy, close their doors against epileptics." (P. 89.) To this dreadful catalogue of irremediable ills other additions have been made.

"Dr. Darwin well remarks, that when an individual becomes insane, who has a small family of children to solicit his attention, the hope of recovery is but small, as it shows the maniacal hallucination to be stronger than those ideas which usually interest us most. And Haslam further states, that in those instances where insanity has been produced by a train of unavoidable misfortunes, as when a father of a large family, with the most laborious exertions, ineffectually struggles to maintain it, the number who recover is very small.

"We are told by Pinel, that religious insanity is for the most part fatal. Haslam also gives the same opinion, as do Cox, Hallaran, and others; I must confess, however, so far as my own observation goes, this is not precisely the case." (P. 90.)

On the other hand, puerperal insanity is commonly cured, and the prognosis is more favourable than the records of hospitals would seem to warrant us in asserting; for, as Gooch observes, the cases of short duration, lasting only a few days or weeks, do not enter the hospital at all. Gooch indeed says, "Of the many patients about whom I have been consulted, I know only two who are still, after many years, disordered in mind; and of these, one had already been so before her marriage." [*On the Disorders of the Mind in Lying-in Women.*]

The seventh chapter, on the "Preventives of Insanity," has some sensible remarks on the gloomy religious theories inculcated into so many children from the very cradle; and there is a letter by a young enthusiast, written during a lucid interval, and giving an account of her feelings, which would make the fortune of a work of fiction. But there is no one who could invent such a letter; it bears the stamp of genuineness. It is taken from Dr. Burrows.

In the eighth chapter, on "Confinement, Classification, Control, and Coercion," we find another discussion on the old point—how much madness makes a madman. Dr. Uwins inclines, and we incline with him, to a favourable view of all doubtful cases; but the doctor's lenity is of a very confined and confining character. "What I have hitherto advanced principally refers to the suing for lunatic commissions;" and by no means militates against sending the half and quarter madmen to Peckham: nay, he cites two cases where the patients asked to be allowed the privilege of being confined. "Here then are two instances; and they are merely selected out of a large number of others, which, but for madhouse guidance and control, would, in all probability, have been cases of confirmed and irremediable insanity; and yet the *madhouse-phobia* continues in a degree to prevail, and will, I again assert, continue to prevail, till madness itself be viewed as a mere disorder, and treated of in the same manner as other incidental deviations from physical integrity." (P. 126.) This *madhouse-phobia* is really a very whimsical disorder: it probably proceeds from an abnormal development of the organ of caution, and can only be cured, we think, by the sufferer's being subjected to what Dr. Uwins calls "the phrenological training of depressing this portion, and pushing out that, of the cerebral mass." (P. 120.) Till this system is adopted, we fear it will "continue to prevail," in spite of the men of Peckham, like so many Circes, spreading out all their allurements to catch us; their novels, newspapers, pianos, steady nurses, and Mr. Norris, who promises, says our author, to be everything that is desirable. Madmen, it seems, are a good deal like schoolboys; and a nervous patient is as much tamed at the sight of a Peckham nurse, as a truant is at the entrance of his master bearing a rod of the most awful dimensions.

"Cruelty is supposed to be exercised within the walls of a lunatic asylum. Than myself I hope there is not a human being who would be more speedy and loud in his condemnation of cruelty; but that such is the case it is more easy to aver than to prove, and I verily believe that, for the most part, tales of injustice and of harsh treatment, deserve much the same kind of credit as that which is to be given to the representations of schoolboys, when they are desirous to rid themselves of the thraldom implied in school discipline. Indeed, dealing with the mad has many points of similarity with that of dealing with the school inmate; and I would state this to be one of the advantages connected with lunatic asylums, or professional management of the insane, that there is less severity needed than when their management is in the hands of relations or common nurses; it is indeed astonishing to find how soon the invalids are made sensible that the custody under which they now are has an authority attached to it, against which it is useless to rebel; but, like the schoolboy, they are much happier in that restraint, provided the power of the keeper be exercised mercifully and with discretion; just as the boy, spoiled during the holidays, feeling, the moment he enters the school-room, the necessity of putting aside all his domestic waywardness and wantonness, finds himself really much more comfortable than when he was the pet and the pest of the drawing-room at home.

"I have just now in recollection the case of an amiable young lady, who was, during a very long period, under my care, and whose parents were desirous of putting off, as long as might be, the application for a nurse from an establishment. One whole night, from mere nervousness, she sat on the floor, moving and waving her hands and body in a strange unmeaning manner, and at the same time making as unmeaning a noise. I now thought it was high time almost peremptorily to urge new kind of management, and from the moment a Peckham nurse entered the room, (who, by-the-way, was a most gentle, kind-hearted, and excellent woman,) all this sort of thing was given up; and, although my patient continued for a very long time highly nervous, we had no repetition of these insane ebullitions." (P. 127.)

We do not think that Dr. Uwins has hit upon a simile very favourable to his own argument: even if we were to reject (which would be very absurd,) all the evidence of schoolboys as to their treatment at school, what should we do with all the evidence that adults ever and anon furnish as to the mishaps of their younger days? Dr. Uwins must surely know that, in vulgar schools, the masters are frequently tyrannical, the provisions coarse, scanty, and unwholesome, and the whole system mean and harassing; while, in our great public schools, the older boys usurp a power which they are totally unqualified to wield, and drive the iron deep into the soul of many an unfortunate victim. Now, unless all evidence save that of keepers and mad-doctors is to be rejected, something resembling these two species of scholastic misrule has been occasionally recognized in lunatic asylums. In those of the humbler class, the proprietor, like a Yorkshire pedagogue, is anxious to squeeze a little profit out of very low terms, and complaints of dirty sheets, or tainted meat, would be called the ravings of insanity. In opulent establishments, on the contrary, the proprietor seldom resides in the house, and the keepers, like the big boys of a great school, taking advantage of his absence, have sometimes been known to gratify both their gayer and their more malignant passions at the expense of the patients; unless indeed all this be a pure fiction of the parliamentary folios.

Dr. Uwins recollects

"an artist, who is as firmly convinced as he exists that spirits are hovering about him with the intention of constantly annoying him; and he sometimes becomes angry with those who are in his company, because they do not, like him, hear the noise of these visitors, which, as he imagines, conceal themselves in the wall. Now who is there hardy enough to say, or wrong-headed enough to think, that this poor man should be sent to engrave in a madhouse, torn from his family and friends, merely because such an alteration had taken place in some part of the brain as to create these visionary conceits, while other parts of the brain retain their wonted power, and his faculties are not interfered with to the extent of doing mischief to himself or others." (P. 128.)

Our author thinks that this man ought not to be torn from his family and friends, and sent to engrave in a madhouse; and he is right: yet he keeps another person at Peckham, who has no symptom of madness, because, if he were let out, "he would be immediately running to taverns, and running up bills, which he would leave to be settled by his poor mother and brother, who are already half ruined by his half-mad and half-criminal conduct. I tell this man, in reply to his petitions for release, that he has only to choose between a madhouse and a jail, in the event of his liberation; but there might be some question as to the propriety of not giving him that choice, had he not been sent to us unequivocally insane, and had he not forfeited his promises of steadiness by his conduct during a probationary release." (P. 133.)

It is very hard if Dr. Uwins cannot fill the Peckham establishment, or Peckham itself, if he allows himself to incarcerate a man whose sole symptom of insanity consists in not paying his bills. But we differ so much from our author as to the propriety and legality of shutting up this unfortunate hotel-haunter, that we would seriously recommend his case to the notice of the parliamentary commissioners.

Dr. Uwins does not like patients to be visited by their friends, more especially when they are convalescent; but he has the candour to quote at full length the well-known case in which Dr. Gooch cured his patient by a single interview, to the unspeakable surprise of Dr. ——, "who came down, saying 'it looks like magic.'" The commissioners, however, absolutely allow visits:

"I say it, then, with respect, but I am bound to say it with boldness, that both as it regards the classification of patients, and the visits of friends, the commissioners under the Act of Insanity, with the most equivocally benevolent designs, have sometimes imposed tasks on the proprietors of lunatic establishments, vexatious to them, and injurious to those who are under their care.

"In thus asserting what I consider important fact, I have no interest, I have had no prompter. 'My withers are unwrung.' I have never asked, never received favours from any man, or any set of men, and I have nothing to expect in life but the fair reward of integrity and industry." (P. 138.)

We are informed by the erratum, that for "equivocally" we are to read "unequivocally; but, as we trust that the commissioners are not only benevolent, (which the emendated reading allows them to be,) but also sensible, we should have been glad to learn what the modicum of visits is which vexes the proprietors, injures the patients, and makes Dr. Uwins talk without a prompter.

As to physical coercion, they do not flog at Peckham; but they use strait-waistcoats, handcuffs, footlocks, and Peckham chairs, which confine both arms and legs, and keep the patient in an upright posture. Some people have imagined that these things might be dispensed with.

"There are some enthusiasts among us, who imagine that improvements in regard to scholastic discipline may be carried so far as to supersede altogether even the mildest plans of punishment; so I verily believe did the more sanguine among those statesmen, who but a few years back were so laudably engaged in the investigation of insanity, and in schemes for the better regulation of insane establishments, conceive that an appeal to the maniac's feeling would prevent altogether the necessity of harsher treatment. Then again, some of our medical reformists lent themselves to the fostering of this false notion; and one of them, I believe, was considerably influenced in his condemnation of madhouses, by the opportunity it afforded him of antithetical and pointed alliteration. 'Stripes and strait-waistcoats'—'Mausolea of mind'—and such like prettinesses figured in his pages, with effective impression, upon those who, like himself, were benevolent theorists, but knew no more of the practical requirements of mental derangement, than a lad who may have been half a year behind the counter of a dispensing shop; and who, of course, in his own estimation, is as fully competent to judge of medical agents as the best of us." (P. 146.)

It is strange that this medical reformist should have affected to be seized with a *madhouse-phobia*, when his real infirmity was rather an *alliteration-philia:* surely, a man of any genius must have seen that alliteration may be adopted on any side of any question. Is not the title of this chapter "Confinement, Classification, Control, and Coercion?"

In chapter ninth, on "Suicide," our author recommends

"contriving some sort of posthumous punishment—not burying in crossroads, certainly—as a warning to others." (P. 152.) There is an old classical story of a general who put a stop to suicides among women, by exposing the bodies of the dead; and Dr. Uwins has a similar anecdote.

"We have, indeed, examples which speak forcibly and unequivocally to the point of prevention. Dr. Burrows speaks of a particular district, I think on the Continent, where, in the army, suicide actually became an epidemic disease; the commander, finding that he lost some of his best soldiers by this unprofitable warfare, ordered the next half dozen who should commit the act to be hung on some neighbouring trees, and exposed to the gaze of their former compeers. The remedy completely succeeded, and the soldiers immediately ceased to play the game of killing themselves. I say the soldiers, but I believe, upon recollection, that the epidemic, although among the military, was not confined to them." (P. 153.)

But why does Dr. Uwins believe it upon recollection? why not consult Dr. B.'s work again? These hesitations and half-recollections are among the graces of an extemporaneous lecture, but destroy the finish of a book. So again, Dr. U. says, (p. 152,) "I am about being led away from the main intention of my present paper;" and, at p. 128, "I am however, in some degree, wandering from my intention of stating, &c.;" and, at p. 55, he actually says, "If I find I have space, I shall take up the subject again, under another division of my treatise." *If I find I have space!*—so that the author, when transcribing cap. iv. for the press, is ignorant of the space occupied by the remaining ones! We know the defence that a modern writer would make: he would allege that the MS. is never rewritten at all; so that the author, when composing p. 55, is bonâ fide ignorant of what is to come at p. 205; and, when he has been "wandering from his intention," cannot spare time to erase his wanderings, and substitute something akin to the point in question. The proof-sheets are then revised in a hurry, continues the defendant, and the work is sent forth into an inhospitable world. What is the consequence? we ask. In six months, nice uncut copies are to be bought for eighteen-pence on the stalls, while others are lining trunks, or sold at miscellaneous auctions under the uninviting title of "sundries."

Chapter the tenth, on the "Moral Management of Nervous Disorders," contains some amusing and instructive narratives of the method in which hypochondriac patients may sometimes be aroused from their melancholy reveries. We will select a couple of these cases.

"I remember a patient of mine, in Buckinghamshire, who having fallen into a nervous or hypochondriac state, yielded to the wishes of an intimate friend, that he would take a journey with him into a distant part of the country, and expose himself to scenes and circumstances of former enjoyment. He complied, as I have stated, with the desire of his companion and other friends; but still, wherever he went, or whatever he saw, was alike indifferent to him. An extinguisher was on his perceptions; he saw, but he did not feel. The individual who travelled with him was about to return to Aylesbury with his melancholy companion, when, on entering a town in the west of England, a sudden shower of rain unexpectedly overtook them. A man on horseback, who was riding in the same direction, had so grotesquely accoutred himself, in the hurry of guarding against a thorough wetting, that the melancholic of whom we are speaking burst into a violent and loud laugh; from that moment he was a well man, and returned to his friends with a countenance cheerful and a mind unburthened." (P. 173.)

"'I had a friend,' says this lively writer, [Mr. Townsend,] 'who was a hard student, buried among his books, in a room of small dimensions, for fourteen hours in the day: he became dyspeptic to a degree that I never witnessed before; his flatulence was so great for three hours every day after he had eaten his dinner, that by this circumstance, independently of natural inclination, he was obliged to live alone. It happened at the same time that this gentleman had a favourite spaniel, who was always at his side. The faithful animal, who should have been ranging in the woods, being thus confined, was afflicted with a deplorable disease, being troubled exceedingly with flatulency, and *borborigmi,* from wind always in motion, and grumbling through the colon.

"'With these symptoms of dyspepsia, poor Rover, for that was his name, from being sprightly, became remarkable for languor, want of energy, and depression of spirits. He was evidently jealous and suspicious; insomuch that, if any called him by his name and spoke kindly to him, he lifted up his eyelids, and slunk away to hide himself: (poor Rover had become of '*unsound mind.*')

"'Happily, at this period, some friends decoyed our student from his books, prevailed on him to get on horseback, to accept of greyhounds, and to go early to the field.

"'Rover followed with reluctance, but, by degrees, they both contracted a fondness for the sport: a long separation took place between our student and his books; and, escaping thus from the occasional causes of debility, whilst he enjoyed the diversions of the field, with fresh air and exercise, on horseback, he lost every symptom of disease; and his faithful Rover, participating in the same diversions, without the assistance of other tonics or astringents, regained his wonted energy: no longer depressed by flatulence and depression of spirits,' (his mind became '*sound*' again.)" (P. 185.)

The eleventh chapter, on the "Medical Treatment of the

Insane," and the last one, which is recapitulatory, do not offer anything particularly worthy of our notice, except perhaps one case of an obstinately silent patient, who was frightened into talking by the galvanic battery.

On the whole, this is an entertaining, and in some measure even an instructive book; though we should conjecture, from the paucity of original cases, that it is founded on a very short practice among the insane.

In spite of numerous barbarisms, such as "the practical requirements of mental derangement" proceeding partly from affectation, and partly from not having had time to revise the sheets, Dr. Uwins' style is fluent, and shows the hand of a practised writer.

